# A Wildfire Smoke Detection System Using Unmanned Aerial Vehicle Images Based on the Optimized YOLOv5

**DOI:** 10.3390/s22239384

**Published:** 2022-12-01

**Authors:** Mukhriddin Mukhiddinov, Akmalbek Bobomirzaevich Abdusalomov, Jinsoo Cho

**Affiliations:** Department of Computer Engineering, Gachon University, Sujeong-gu, Seongnam-si 13120, Republic of Korea

**Keywords:** wildfire detection, forest fire, smoke, UAV images, YOLOv5, deep learning, CNN

## Abstract

Wildfire is one of the most significant dangers and the most serious natural catastrophe, endangering forest resources, animal life, and the human economy. Recent years have witnessed a rise in wildfire incidents. The two main factors are persistent human interference with the natural environment and global warming. Early detection of fire ignition from initial smoke can help firefighters react to such blazes before they become difficult to handle. Previous deep-learning approaches for wildfire smoke detection have been hampered by small or untrustworthy datasets, making it challenging to extrapolate the performances to real-world scenarios. In this study, we propose an early wildfire smoke detection system using unmanned aerial vehicle (UAV) images based on an improved YOLOv5. First, we curated a 6000-wildfire image dataset using existing UAV images. Second, we optimized the anchor box clustering using the K-mean++ technique to reduce classification errors. Then, we improved the network’s backbone using a spatial pyramid pooling fast-plus layer to concentrate small-sized wildfire smoke regions. Third, a bidirectional feature pyramid network was applied to obtain a more accessible and faster multi-scale feature fusion. Finally, network pruning and transfer learning approaches were implemented to refine the network architecture and detection speed, and correctly identify small-scale wildfire smoke areas. The experimental results proved that the proposed method achieved an average precision of 73.6% and outperformed other one- and two-stage object detectors on a custom image dataset.

## 1. Introduction

When wildfires occur, the first thing observed in the air is a massive column of smoke. A reliable smoke alarm is essential for preventing fire-related losses. Rapidly spreading wildfires, exacerbated by climate change, can have far-reaching consequences on human communities, ecosystems, and economies, if not detected or extinguished quickly [1,2]. Smoke and flame detection are both applicable to wildfire monitoring. Smoke is the first visible indicator of a wildfire. Therefore, an early warning wildfire detection system must be able to detect smoke in natural environments. Smoke from wildfires has the following three primary properties: it is physically present, visually distinct, and dynamic. For a sensor to collect representative samples of smoke, it must be within close proximity of the smoke to detect its physical characteristics. In this study, we primarily focus on the other two properties, visually distinct and dynamic, which are perceptible to a camera. Initially, this section describes the economic issues and the reasons for the wildfires. Second, we analyze the current sensors and methods to detect wildfire smoke. Following this, a deep learning (DL) approach for detecting wildfire smoke is proposed.

Recently, many wild areas and forests have been burned or destroyed. Every year, wildfires destroy millions of acres of land, resulting in enormous losses to human life, vegetation canopies, and forest resources [3,4]. Wildfires are uncontrollable natural disasters that seriously threaten national economies. In addition, dry soil and crop destruction caused by fires have a negative effect on agricultural activities and crop productivity in areas closest to the blazes. The United States, Australia, Brazil, and Canada are just a few countries with a history of devastating wildfires [5,6,7,8]. More than 1500 buildings were destroyed in the recent Australian wildfire, along with approximately half a million animals and 23 people. Overall, more than 14 million acres have been gutted by fire [9]. The California fire of 2018 and the Amazon rainforest fire of 2019 were two examples of destructive wildfires that caused significant losses and burned millions of acres of land [10]. Two recent studies confirmed that climate change is a primary factor in wildfires [11,12].

As an alternative to direct wildfire detection, smoke detection can be used to determine the presence of fires owing to the following properties of wildfire smoke: (1) the temperature of fire smoke is remarkably lower than that of the hot ground; (2) fire smoke can rise above the canopy in a short amount of time and usually has specific colors from the vegetation, making it easier to detect from unmanned aerial vehicles (UAV); (3) fire smoke spreads faster and on a larger scale than the fire; thus, it is more straightforward to detect from UAV. However, satellite-based fire smoke detection encounters several obstacles. Fire smoke shares similar and overlapping spectral signatures with a wide variety of other objects, such as fog, haze, snow, clouds, dust, and plants. Smoke always appears far from the camera, and the area of smoke typically accounts for only a small portion of the video frame [13,14,15,16,17].

Point-based sensors have traditionally been used to detect fires [18]. These sensors collect data by taking readings of the surrounding environment, such as the temperature and humidity, as well as by sampling smoke particles. Conventional fire sensors have been widely adopted owing to their low cost, ease of use, and reliability. However, there are certain problems with these sensors that are difficult to fix. To analyze the particles, temperature, or humidity, these sensors must take direct samples of the combustion products. Therefore, these sensors should be placed in close proximity to the potential fire sources. Consequently, the use of such sensors in contained or indoor settings is restricted. Additionally, it may take some time for combustion products, such as smoke particles, to be transferred to the sensors, resulting in a delayed response.

Vision-based fire detection has been extensively researched owing to the aforementioned conventional methods [19]. Through the investigation of fires, we learned that smoke typically travels more rapidly than the fire itself. Therefore, smoke detectors can sound off earlier than flame detectors in the event of a fire. Traditionally, cameras were placed on watchtowers to monitor potentially hazardous areas and spot fires as soon as they broke out. Furthermore, people would monitor multiple screens simultaneously to spot the signs of a wildfire early on. Over the past few decades, numerous methods for detecting wildfires have been proposed to aid monitoring. The most noticeable quality of the smoke is its color. For this reason, much emphasis has been placed on different color spaces for smoke detection [20,21].

Recently, promising technologies for wildfire monitoring have emerged, such as UAV-based early wildfire detection and warning systems that integrate various remote sensing technologies and DL-based computer vision techniques [22,23]. To monitor and combat wildfires, UAVs equipped with visual remote sensing technologies can be used instead of sending ground forces into hazardous circumstances or using various traditional techniques with several limitations in terms of efficiency, accuracy, and price. Effective communication technologies, such as LoRaWAN and 5G [24], combined with UAVs, can detect smoke early, sending valuable information to concerned authorities. The typical process of creating UAV image-based fire-identification technology involves analyzing images for a specific color, motion, and geometric features [25]. In recent years, numerous DL-based smoke- and fire-detection algorithms have been proposed, each with promising results. Most existing wildfire smoke detection algorithms are based on convolutional neural networks (CNNs). These include YOLO [26,27], R-CNN [28,29], SSD [30], U-Net [31], and DeepLab [32]. Other deep learning (DL) architectures, such as long short-term memory (LSTM) [33], deep belief network [34], and generative adversarial network (GAN) [35], can also be used for fire detection. However, the real-time execution of such algorithms requires high-power hardware. Recent advancements in computing power, sensing devices, and development software have allowed the use of UAVs for wildfire smoke detection using sophisticated DL-based computer vision algorithms. UAVs can quickly locate an issue, pinpoint its precise location, and alert the appropriate authorities. Hu et al. [36] proposed the MVMNet model for the accurate detection of targets in wildfire smoke. To extract color feature information from wildfire images, Guan et al. proposed the FireColorNet model based on color attention [37]. Fan et al. [38] proposed a lightweight network architecture for wildfire detection. YOLOv4-Light replaces YOLOv4’s core feature extraction network with MobileNet and path aggregation network (PANet)’s regular convolution with a deep separable deep convolution. Federico et al. [39] established a faster R-CNN model for object detection and developed a DL model for detecting forest fires using the transfer learning of a pre-trained RetinaNet. However, UAV images captured from the above model cause target wildfires to appear tiny, with color and shape features that are not immediately visible, making early detection difficult. Thus, the aforementioned color- and shape-feature-based wildfire detection models cannot be directly applied to UAV images. The research on UAV-based methods for the early detection of wildfires faces significant challenges. Furthermore, the precision of wildfire detection based on UAV images is negatively affected by the deficiency of labeled UAV fire image samples. However, introducing DL methods into UAV image recognition is challenging because of the lack of sufficient fire annotation image samples; such methods require large amounts of high-quality annotation data to achieve satisfactory detection results.

Constructing a deep-level network model is required to extract more abstract features from images; however, training a deep neural network is a time-consuming and challenging approach. Furthermore, a large number of labeled samples are required to train a deep model. Consequently, this has become a significant bottleneck in identifying wildfires from UAV images; however, recent advances in transfer learning offer hope for a solution. Transfer learning [40,41] describes the application of a trained model to a different task and then using that model to model the new task by adjusting its parameters to better suit the new context. Overfitting training owing to insufficiently labeled samples can be avoided with transfer learning.

We propose a wildfire smoke detection and notification system based on the enhanced YOLOv5m model [42] and UAV images, which can help overcome the above-mentioned limitations. To identify smoke from wildfires, a core framework that was pre-trained using the common objects in context (COCO) dataset was used. The original network was enhanced by optimizing the network structure parameters, and the pre-trained weights were used as initialization parameters for the backbone network. Noxious gases can be accurately identified by applying the optimized network to the wildfire smoke dataset. Our previous findings [43] inspired this study. As described in Section 3 and Section 4, we enhanced the performance of the traditional YOLOv5m network to facilitate rapid wildfire smoke detection and tested our findings on an artificial intelligence (AI) server. The main contributions of this study are as follows:

- A fully automated wildfire smoke detection and notification system was developed to reduce natural catastrophes and the loss of forest resources;
- A large wildfire smoke image dataset was collected using UAV and wildland images of wildfire smoke scenes to improve the accuracy of the deep CNN model;
- Anchor-box clustering of the backbone was improved using the K-mean++ technique to reduce the classification error;
- The spatial pyramid pooling fast (SPPF) layer of the backbone part was optimized to focus on small wildfire smoke;
- The neck part was adjusted using a bidirectional feature pyramid network (Bi-FPN) module to balance multi-scale feature fusion;
- Finally, network pruning and transfer learning techniques were used during training to improve the network architecture, detection accuracy, and speed.

The remainder of this paper is organized as follows: Section 2 describes the literature on UAV- and DL-based wildfire smoke detection methods. The experimental dataset is presented in Section 3, along with a comprehensive analysis of the structure of the YOLOv5 model. Section 4 provides an in-depth discussion and an analysis of the experimental findings. Analysis of wildfire smoke detection based on various systems is discussed in Section 5. The shortcomings of the proposed system are addressed, and future directions are outlined in Section 6. Finally, Section 7 summarizes this work.

## 2. Related Works

Several vision-based methods have been proposed for wildfire smoke detection, the most prominent of which are image color and deep CNN. In addition to recent successes of DL in natural language processing [44] and image classification [45], significant progress has also been made in DL-based wildfire detection methods.

### 2.1. Conventional Image-Based Methods

Wildfire smoke is the most prominent characteristic of early-stage fire-detection systems. Early smoke detection has been a focus of interest for some researchers. Smoke color information was extracted using the energy function by Calderara et al. [46], allowing for wildfire smoke detection. Their camera-based system was adequate for detecting fires in an area of 80 m^2^ during the day and night. Nevertheless, color-based smoke detection is not robust because certain smoke colors, such as black, gray, and white, are similar to background environments, including clouds, dust, and mountains. Some researchers have combined color, texture, and dynamic features to enhance smoke detection implementation [47]. Ye et al. [48] employed a pyramid approach to conducting multi-scale decomposition of smoke images and merged it using a support vector machine (SVM) to achieve smoke identification. This method improves the wildfire detection precision because it simultaneously considers the spatial and temporal information of the image sequences. Ye et al. [49] utilized the common motion characteristics of fire and smoke to identify the smoke. At the same time, Islam et al. [50] used color and motion to identify smoke using a combination of Gaussian mixture model (GMM)-based adaptive moving object detection and an SVM classifier. Their method achieved 97.3% accuracy but did not help detect accidental fires beyond the range of surveillance cameras. Previous color-based fire detection methods require extensive parameter tuning, which negatively affects detection stability. To lessen the importance of fine-tuning, Khalil et al. proposed a new fire detection method using the RGB and CIE L*a *b color models by integrating movement detection with tracking fire regions [51]. Their technique relied on a GMM that utilized segmented images of fire to identify only the motion of objects that matched the color of the fire. This step only detects moving fire pixels and ignores the other moving pixels.

### 2.2. Deep Learning and UAV-Based Wildfire Smoke Detection

In recent years, there has been an increase in the use of UAVs for a wide range of forestry tasks, such as exploration and saving procedures, forest scouting, forest firefighting, and forest resource surveys. Thus, they represent one of the most promising novel approaches for addressing the issue of wildfire smoke detection. Therefore, owing to their high flexibility, low price, ease of use, and ability to fly at various heights, UAVs systems are preferred over other available technologies. Owing to developments in hardware and software, it is now feasible to process intensive visual data directly from UAV. Interest in using deep learning-based computer vision techniques for detecting fire and smoke in forests and wildland areas has recently increased. UAVs can employ deep-learning algorithms to autonomously identify the origins of wildfires based on the following two key visual features: smoke and fire. Smoke and fire are the most useful visual cues for quickly and accurately detecting wildfires. Some researchers have concentrated on wildfire detection through fires [52,53]. In contrast, other studies have focused on wildfire detection using smoke features [54,55], which appear to be more appropriate for early wildfire detection because the fire in its early scene could be concealed, particularly in overgrown forests [56,57]. Several recent studies have aimed to simultaneously detect smoke and fire. Early wildfire detection using UAV and deep learning algorithms can be achieved in the following three main ways: wildfire image classification, wildfire detection based on object detection algorithms, and semantic segmentation-based wildfire detection. There are the following three primary ways to accomplish early wildfire detection using UAV and deep learning methods: wildfire detection based on object detection approaches, wildfire image classification, and wildfire detection using semantic segmentation [58]. However, training these methods requires a significant visual dataset and robust computational resources, such as hardware and software. We also need to be careful when selecting an appropriate network architecture and training it using an appropriate dataset. In Section 3, we explain and present the proposed early wildfire smoke detection system using deep learning techniques.

## 3. Materials and Methods

**Figure 1:**
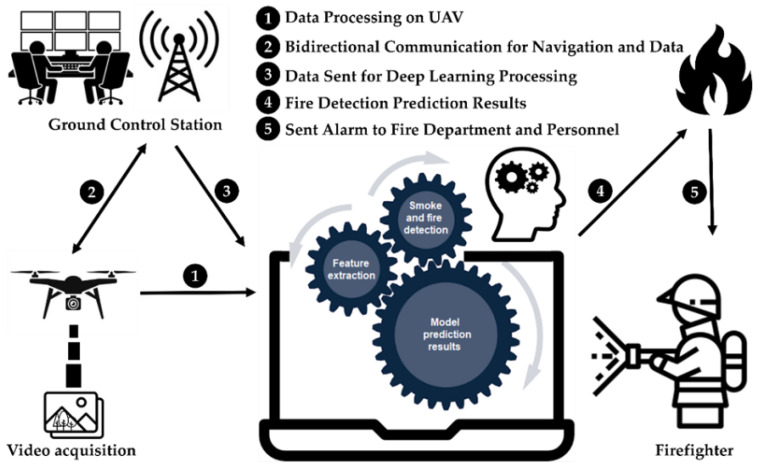
Overall framework of the UAV-based wildfire smoke detection system.

In this section, we explain the deep learning model used for wildfire smoke detection tasks, the dataset used for training, and the evaluation metrics employed in this study. Prior to the beginning of the task, the navigation procedure, selection of suitable models and algorithms, and the execution of the system must be completed. As shown in Figure 1, the UAVs camera is used to take photos or videos, and the computer performs preprocessing, feature extraction, smoke, and fire detection, and generates prediction results.

### 3.1. Overview of the UAV-Based Wildfire Detection System

This study used UAV images, computer vision, and deep learning models to enhance the precision of early wildfire smoke detection results in cloudy, hazy, and sunny weather conditions. We propose an optimized YOLOv5 model and UAV image-based wildfire smoke detection and notification system. Typically, UAVs are equipped with cameras that send data to a ground control station, which is analyzed using an AI system to detect the presence of smoke or fire. The proposed system employed deep CNNs to detect smoke regions with high accuracy and a strong processor to execute quick real-time image processing.

Figure 1 shows the overall framework of the UAV-based wildfire-smoke detection system. Section 3.2 gives a more detailed explanation of the proposed system. Section 3.3 describes data collection and preprocessing, while Section 3.4 and Section 3.5 explain transfer learning and evaluation metrics. In this study, we focused on developing an AI system for early wildfire smoke detection and compared its performance with that of YOLO models and other state-of-the-art methods.

When working with a UAV, it is essential to control and receive image and video data remotely. Therefore, the life-of-sight, 4G/LTE, and SATCOM communication methods were used to secure the capability of operating under various circumstances and the UAV operation at long distances from the ground control station due to the size of the forest area. A typical transmission structure contains a line-of-sight ground control station using a radio connection. It includes two datalinks (the primary one, used for image and video and telemetry exchange within 180+ kilometer range, and the backup one, for telemetry only), with automatic hopping between them in case of Global Navigation Satellite System (GNSS) or signals loss and advanced encryption standard AES-256 encryption. Secure VPN technologies, including TLS, IPSec, L2T, and PPTP, are used for data transport. This method allows the ground control station to connect with the UAV regardless of range restrictions and provide reliable cellular service. The modem concurrently enrolls itself in the networks of two distinct cellular network operators and then chooses the most reliable one. Line-of-sight communications have some disadvantages, considering the range and the possibility of weather interference. SATCOM has historically been considered a Beyond Line of Sight (BLOS) communication system that would guarantee a constant connection and reliable data transmission at predetermined distances. A highly directed L-band antenna ensures a small radio signature. Furthermore, it complies with BRENT, STU-IIIB, TACLANE, STE, and KIV-7 are only some of the encryption and secure communication standards. AI server computer is located in the ground control station to process received image and video data from UAVs.

The framework presented in Figure 1 is the fundamental procedure for detecting smoke from wildfires. The deep learning methods applied in this procedure have significantly facilitated the operations of feature extraction and detection by substituting traditional approaches [59].

**Figure 2:**
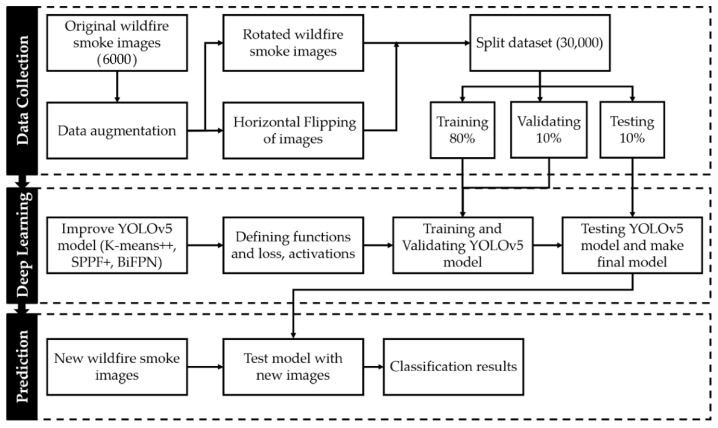
The flow chart of the proposed method.

After acquiring the image and performing the necessary optimization procedures during preprocessing, it is necessary to isolate pixels that describe the object of interest from the rest of the image. Smoke and fire feature extraction consisted of images taken at specific times of the day and with specific lighting conditions. Motion, colors, corners, edges, brightness levels, and intensities are image characteristics that were considered in the feature extraction process. To perform an in-depth analysis of the segmented image and locate the essential points of interest, the image was feature extracted, which means that the relevant operations are being executed on it. The resulting image was then fed into a trained model to locate patterns that will either validate or invalidate the existence of smoke. Figure 2 depicts the detailed procedure of the proposed approach. In the subsequent step, if the AI model produced a positive result, the system sends an alarm via the UAV or the ground support station to the personnel responsible for fire protection to take the necessary steps.

### 3.2. Proposed Wildfire Smoke Detection Method

In this section, we discuss the procedures of computer vision techniques based on deep learning that are executed on an AI server to detect smoke from wildfire images. Our strategy consisted of developing several computer vision techniques that use deep learning to accomplish our purposes.

#### 3.2.1. Original YOLOv5 Model

A state-of-the-art real-time one-stage object detector, YOLOv5, is well suited to our needs, owing to its shorter inference time and higher detection accuracy. Because of its developers’ commitment to improving the system, YOLO has become one of the most effective methods for detecting objects in both Microsoft COCO datasets and Pascal VOC (visual object classes). YOLOv5 consists of the following four main models: the extended YOLOv5x, benchmark YOLOv5l, and simplified preset models YOLOv5s and YOLOv5m. The primary distinction between these types of networks lies in the number of feature extraction modules and convolution kernels present at various nodes in the network, with a reduction in resulting model sizes and parameter counts.

**Figure 3:**
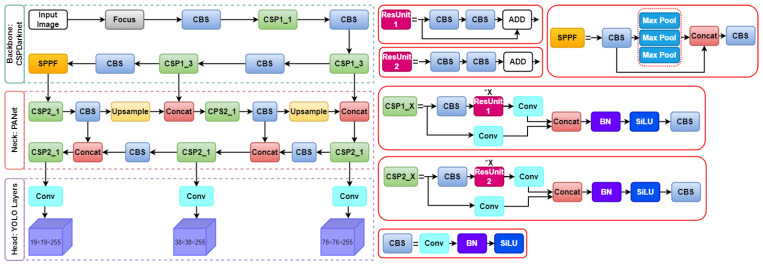
The network architecture of original YOLOv5 model.

Figure 3 illustrates the overall network architecture of Yolov5’s system. The YOLOv5 model can be broken down into the following three primary parts: backbone, neck, and head. First, Cross-Stage-Partial (CSP) 1 and CSP2 were created for CSPNet [60] and had two distinct bottleneck CSP structures. The goal is to decrease the amount of duplicated information. Consequently, the model parameters and the number of floating-point operations per second (FLOPS) were scaled back. This has the dual effect of accelerating the inference process while simultaneously improving its precision, leading to a smaller mode size. Among them CSP1—also known as backbone— and CSP2—also known as neck— were used for feature fusion. Both processes are described below. Second, in addition to CSP1, the backbone features Convolution Layer + Batch normalization + Sigmoid Linear Unit (CBS) and spatial pyramid pooling fast modules. The spatial pyramid pooling fast module chained three 5 × 5 MaxPool layers together, iteratively processed the input through each layer, and finally performed a Concat on the combined output of the MaxPools before applying a CBS operation. Spatial pyramid pooling fast is faster than spatial pyramid pooling while producing the same results. Third, Neck employed a PANet [61]. Using an improved bottom-up path structure, PANet employed a new feature pyramid network (FPN) to transfer feature information at the lowest possible level.

Furthermore, adaptive feature pooling, which connects feature grids to all feature levels, directly reproduced valuable data within each feature level in the following layer. Thus, PANet can use precise localization signals to enhance the precision with which objects are located in the lower layers. Finally, the head enabled the model to predict the size of objects across various scales by the generation of three feature maps, one each for small, medium, and large objects.

#### 3.2.2. K-Means++ Clustering Technique for Determining Anchor Boxes

In object-detection approaches, high-precision detection requires an appropriate anchor box. Anchor boxes are a set of initial regions that share fixed dimensions and aspect ratios. The easier the model can be trained, the closer the predicted boundary box aligns with the actual boundary box and the closer it is to the actual boundary box. Consequently, the anchor parameters of the original YOLOv5 model must be modified to meet the needs of specific datasets during training. K-means clustering has been used in the field of clustering owing to its simplicity and efficiency. K-means clustering was used in the YOLOv5 model to obtain the k initial anchor boxes. This method requires artificially setting the initial clustering centers, which can lead to noticeable differences in the final clustering output. The main drawback of the K-means algorithm is that it requires inputs, such as the initial clustering center and the number of clustering centers, *k*. However, specifying the exact locations of the clusters and the initial cluster center in advance is notoriously problematic. In this study, the K-means++ algorithm was used to obtain k initial anchor boxes, which fixes the problems with the original K-means algorithm. To obtain an anchor box size better suited for detecting small objects, K-means++ optimizes the initial point selection and can thus significantly reduce the classification error rate.

The following is a detailed explanation of how K-means++ was used to find an anchor box:

(1) Select a random central coordinate *c_1_* from the given dataset X;

(2) Determine the Euclidean square distance $D_{\left(x\right)}$ between each sampling point and center;

(3) Compute the probability of each sampling point $P_{\left(x\right)}$ to serve as a new cluster center. The sampling point with the maximum probability was chosen to serve as the center of the new cluster. The probability was calculated using the following formula:

(1) $$P_{\left(x\right)}=\frac{D_{{\left(x\right)}^{2}}}{\sum_{x\in X}D_{{\left(x\right)}^{2}}}$$

where $D_{\left(x\right)}$ is the distance in Euclidean space, and each data point in the dataset has to the cluster’s center. Each point in the sample has a certain chance, denoted by $P_{\left(x\right)}$, of becoming the next cluster’s epicenter;

(4) Once we have selected the center of the first K clusters, repeat Steps (2) and (3). We define *C* as the set of closest points and revise the mass center for each set of *C* for *i* ∈ {1, 2, 3,..., k};

(5) Repeat step (4) until the value of *C* does not change by more than the threshold, or the maximum number of iterations is reached.

#### 3.2.3. Spatial Pyramid Pooling Fast

**Figure 4:**
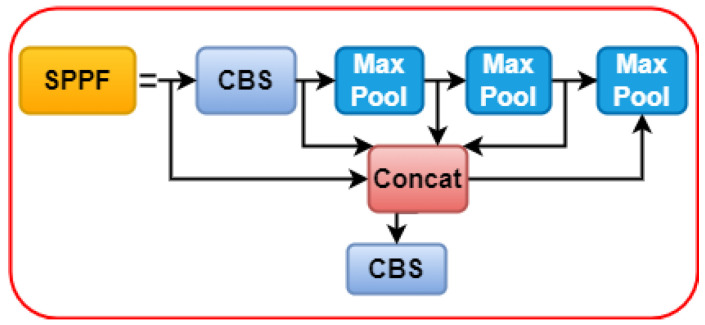
The flowchart of spatial pyramid pooling fast.

The newest version of YOLOv5, 6.1, includes the spatial pyramid pooling fast (SPPF) as the final module of the backbone. The SPPF module comprises three 5 × 5 MaxPool layers through which inputs are iterated; the combined output of the layers is then concatenated before the CBS operation is performed. Figure 4 shows a flowchart of SPPF. The image can learn features at multiple scales with the help of maximum pooling and jump connections, and then combine global and local features to increase the representativeness of the feature map. Maximum pooling is a method that uses a rectangular mask to extract the maximum value from a set of image regions. Although maximum pooling can help reduce irrelevant data, it often results in the discarding of less useful feature data.

**Figure 5:**
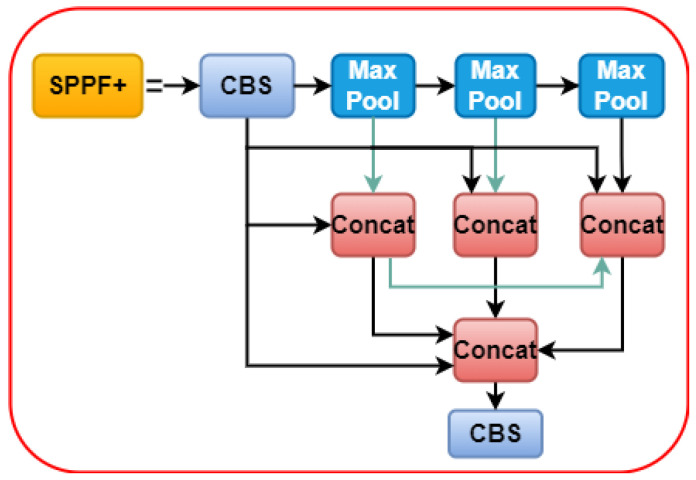
The flowchart of spatial pyramid pooling fast+.

This study enhances the concept of feature reuse while simultaneously enhancing SPPF by utilizing a dense link construction similar to that of DenseNet [62]. Subsequently, we obtain the SPPF module, which helps minimize the feature information lost owing to maximum module pooling. The SPPF+ module effectively retains global information on fires affecting small target forest areas. A flowchart of SPPF+ is shown in Figure 5.

#### 3.2.4. Bi-Directional Feature Pyramid Network

**Figure 6:**
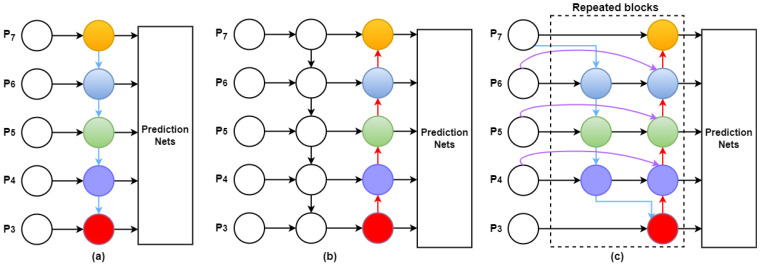
Structures of FPN, PANet, and Bi-FPN: (**a**) FPN [64] presents a top-down pathway (**b**) PANet [61] adds an extra bottom-up pathway on top of FPN; (**c**) Each top-down and bottom-up path is considered as a separate layer in Bi-FPNs feature network. The same layer is repeated multiple times to facilitate more sophisticated feature fusion.

The goal of multiscale feature fusion is to combine features from various scales [63]. Typically, given a list of multiscale features, $P^{in}=\left(P_{l_{1}}^{in},P_{l_{2}}^{in},\ldots \right)$, where $P_{l_{i}}^{in}$ denotes the feature at level $l_{i}$. The purpose is to discover a conversion *f* that successfully gathers various features and produces a list of new features, $P^{out}=f\left(P^{in}\right)$. Figure 6a shows a concrete illustration of a conventional top-down FPN [64]. Input features $P^{in}\;$ is obtained from the backbone network, which is CSPDarknet as shown in Figure 2. It accepts level 3–7 input features $P^{in}=\left(P_{3}^{in},\ldots P_{7}^{in}\right)$, where $P_{i}^{in}\;$ describes the feature level of the input image with a size of $1/2^{i}.$ For example, if the input size is 640 × 640, then $P_{3}^{in}$ corresponds to feature level 3 (640/$2^{3}$ = 80) with a size of 80 × 80, whereas $P_{7}^{in}$ corresponds to feature level 7 with a size of 5 × 5. The traditional FPN aggregates multiscale features from the top down as follows:

(2) $$P_{7}^{out}=Conv\left(P_{7}^{in}\right)$$

(3) $$P_{6}^{out}=Conv\left(P_{6}^{in}+Resize\left(P_{7}^{out}\right)\right)$$

(4) $$P_{3}^{out}=Conv\left(P_{3}^{in}+Resize\left(P_{4}^{out}\right)\right)$$

where Resize is typically an upsampling or downsampling operation for matching the size, and Conv is a convolutional operation for feature extraction.

Only top-down data flow naturally restricts the traditional FPN multiscale feature fusion. Figure 6b illustrates how PANet [61] addresses this issue by adding a bottom-up direction aggregation network.

When using the Bi-FPN as the feature network, top-down and bottom-up bidirectional feature fusion is frequently applied to the level 3–7 features (P3, P4, P5, P6, P7) obtained from the backbone network. The box and class networks receive these combined features as input and output predictions for the class of the object and bounding box. As described in [65], the weights of the box and class networks were cumulative across all feature levels. Figure 6b,c show the feature network designs of PANet and Bi-FPN, respectively.

The use of Bi-FPN to enhance YOLOv5’s neck facilitates a more accessible and quicker multiscale feature fusion. Bi-FPN can also regularly apply top-down and bottom-up multiscale feature fusion owing to the introduction of learnable weights. Compared with Yolov5’s neck PANet, Bi-FPN performs better with fewer parameters and FLOPS, without sacrificing accuracy. This enhanced the capability of identifying wildfire smoke in real time.

#### 3.2.5. Network Pruning

There are the following two primary types of pruning techniques: unstructured and structured. To obtain a particular proportion between the model’s performance and the number of parameters, unstructured pruning employs techniques, such as kernel-level pruning, vector-level pruning, and fine-grained pruning. This type of pruning technique suffers from the need for a dedicated algorithm to support it whenever the network topology changes. When performing structured pruning, the pruning technique primarily modifies the total number of feature channels and filter banks of the network. By contrast, structured pruning can successfully prune an entire network layer without requiring a custom-designed algorithm. In this study, structured pruning was employed to refine the architecture of an improved YOLOv5m network.

The YOLOv5 network architecture comprises three detection heads formed by a cascade of three different forms at the neck. The three detection heads have different output feature scales (19 × 19, 38 × 38, and 76 × 76) and are used to detect small, medium, and large objects in the images, respectively. There is a need to increase both the efficiency and precision of the detection of small-sized smoke. However, because of its time-consuming nature, the 76 × 76 detection head is not well suited to boosting the inference speed. Thus, a structural pruning technique was applied to the YOLOv5 network’s neck part, which involved removing the large object detection heads (76 × 76) and leaving only the medium and small object detection heads in place (38 × 38 and 19 × 19).

### 3.3. Wildfire Smoke Detection Dataset

**Figure 7:**
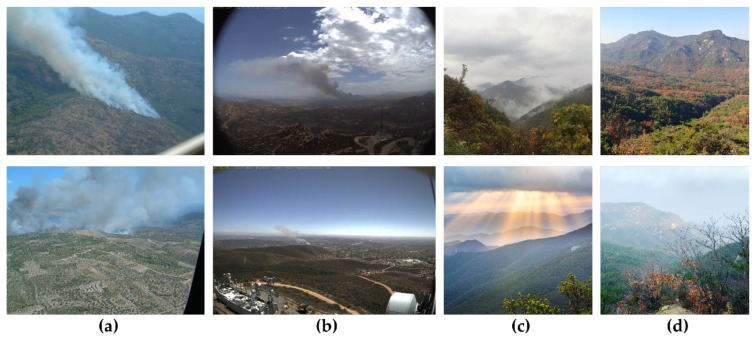
Sample image of wildfire smoke dataset. (**a**) Large and medium size smoke images, (**b**) small-size smoke images, (**c**) non-smoke such as fog and cloud images, (**d**) non-smoke wildland images.

The accuracy of the deep learning model was highly dependent on the datasets used during the training and testing stages. Our analysis of wildfire smoke detection datasets revealed that the datasets created for vision-based wildfire smoke detection systems were deficient and that existing open-access datasets had their own set of problems. Existing wildfire UAV images [66], a Korean tourist spot database [67] for non-wildfire mountain images, and the Kaggle, Bing, Google, and Flickr images were used to address these concerns. Both datasets were crawled from images or videos obtained using a drone, as the early wildfire smoke detection model is meant for applications in drones and UAVs for monitoring. Collected images mainly include aerial pictures of wildfire smoke and aerial images of forest backgrounds. The resolution of collected pictures varies between 2048 × 3072 and 512 × 512 pixels. Recent wildfires in Australia and California are depicted in these images, along with images from Alberta, British Columbia, Colorado, North and South Carolina, and Indonesia, among others. The dataset sample is shown in Figure 7. The dataset consisted of 3285 wildfire smoke images and 2715 non-wildfire smoke images, which were resized to 640 × 640 resolution for network input, as presented in Table 1.

**Figure 8:**
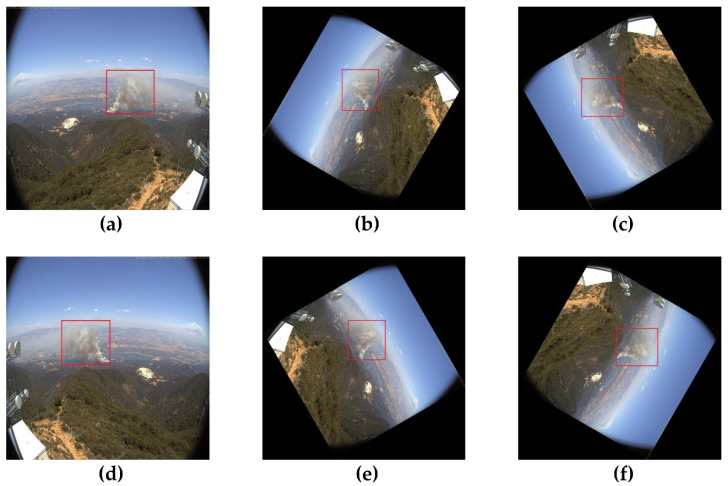
Image data augmentation using geometric transformations: (**a**) original image; (**b**) 60° rotation; (**c**) 120° rotation; (**d**) horizontal flipping of original image; (**e**) horizontal flipping of 60° rotated image; (**f**) horizontal flipping of 120° rotated image.

The success of any deep learning model relies heavily on the availability of a large amount of labeled training data. Nevertheless, reliable results for wildfire smoke detection are difficult to achieve in practice, using this dataset. This could be because of insufficient data, class imbalance, or overfitting. Overfitting a model makes it impossible to capture visual patterns accurately. We used image data augmentation (modifying and reusing images) to increase the predictive ability of the model because insufficient data can cause underfitting. After reviewing the literature [68,69], we found that geometric transformations, such as flipping and rotation, are the most effective techniques for image data augmentation and conducting experiments [70,71]. The efficacy of CNN models depends on the size and resolution of the image datasets used to train the models. Consequently, we applied data augmentations such as rotation, horizontal flip, and the mosaic method to increase the number of images in the wildfire-smoke detection dataset. As shown in Figure 8, we performed the following transformations on each original fire image: 60° and 120° counterclockwise rotations and a horizontal flip. Therefore, we squeezed the preexisting training images to make them more generalized, enabling the model to acquire knowledge from a wider variety of scenarios. The time required to manually rotate and label every dataset image was substantial. We developed a software that uses the OpenCV library to automatically rotate and flip images to simplify the image transformation procedure.

The smoke coordinates shift when the labeled images are rotated counterclockwise at specific angles. We developed a software to read the images in the folder, rotate them, and update their labels so that we would not have to relabel them manually. All wildfire smoke in the images was labeled using the LabelImg tool per YOLOv5 training annotation. The location of smoke was recorded in a text file within the tag folder. This was also implemented in the CNN training process. To reduce the number of false positives, we also included training images that did not depict smoke but were similar, including wildlands, fog, and clouds, as shown in Figure 7.

The 6000 images used for wildfire smoke detection were split into training and test sets, with 80% (4800) being used for training. After applying the data augmentation techniques to the training set only, we expanded the dataset images five times. According to Table 2, the total number of images for wildfire smoke detection has expanded to 30,000.

### 3.4. Transfer Learning

A deep neural model requires several samples to train the model to be effective. It is challenging to obtain good detection results by training from scratch because the initial wildfire smoke dataset is part of a small example dataset. In contrast to fine-tuning, which entails using a portion of a network that has already been trained on a known dataset to train a new unseen dataset, transfer learning involves applying previously acquired knowledge from one domain to a new unseen domain. The trained model served as a baseline for training the target dataset.

**Figure 9:**
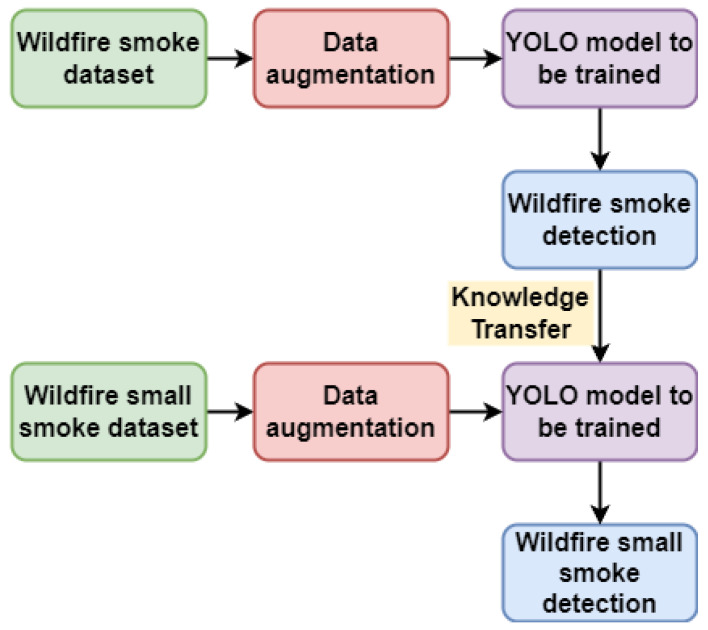
Transfer learning process of training the small-sized wildfire smoke detection model.

In this study, we trained a model to detect small-scale wildfire smoke using a transfer-learning approach to increase the precision of the model. The wildfire smoke detection model was obtained by training on the wildfire smoke dataset and then using that model to train on the small-sized wildfire smoke training set to obtain the small-size wildfire smoke detection model. The training of the small-sized wildfire smoke detection model using transfer learning is illustrated in Figure 9.

### 3.5. Evaluation Metrics

Based on our previous studies [41,72,73,74], we conducted quantitative experiments using Microsoft COCO benchmarks (Table 3), which are commonly used in object detection tasks, and analyzed the results. The precision of a classifier can be measured by the number of correct identifications it makes or the number of times it correctly identifies an object. The ability of a model to recognize important cases is quantified by its recall, which is the rate of correct predictions relative to the total number of ground truths. Good models have a high recall (the ability to identify most ground-truth objects correctly) while recognizing only the objects of interest (it shows high precision). If the recall and precision of a model are both 1, then the model is perfect, and the false-negative value is zero. The accuracy and recall rates were calculated by comparing the results of the proposed method with the ground-truth images at the pixel level. The precision and recall of the wildfire smoke detection system were determined using the following equations:

(5) $$Precision_{C_{ij}}=\frac{TP_{C_{ij}}}{TP_{C_{ij}}+FP_{C_{ij}}},$$

(6) $$Recall_{C_{ij}}=\frac{TP_{C_{ij}}}{TP_{C_{ij}}+FN_{C_{ij}}},$$

where *TP* represents the number of smoke regions that were correctly identified, and *FP* represents the number of false positives that occurred when non-smoke regions were misidentified as smoke. *FN* represents the number of false negatives that occurred when the actual smoke region was incorrectly identified as a nonsmoke region. Based on Equation (7), we determined the average precision (*AP*) as follows:

(7) $$AP_{C_{ij}}=\frac{1}{m}\sum_{j=1}^{m}Precision_{C_{ij}},$$

## 4. Experimental Results

This section explains the experiments conducted and the results of the AI server smoke-detection models for wildfires. The enhanced YOLOv5m model was trained on a personal computer equipped with an 8-core 3.70 GHz CPU, Nvidia GeForce 1080Ti GPUs, and 32 GB of RAM [41]. A wildfire smoke dataset was used for training and testing. The width and height of the input image were 640 × 640 pixels, the number of epochs was 600, the subdivision was 8, the learning rate was 0.001, and the batch size was 32. To enhance the model’s performance, we modified the following particularly crucial parameters: batch size and learning rate.

A high-performance AI server was selected over embedded systems to improve the energy storage activity of UAV systems and guarantee real-time system implementation. The effectiveness of the proposed system in detecting smoke from wildfires depends on the efficiency of the AI server. There is a high demand for AI server processing power to train deep-learning models for smoke detection and notification systems in the event of wildfires. Table 4 shows the results of our experiments, in which we evaluated the implementation of the proposed system with the help of a high-power AI server [41].

### 4.1. Qualitative Evaluation

**Figure 10:**
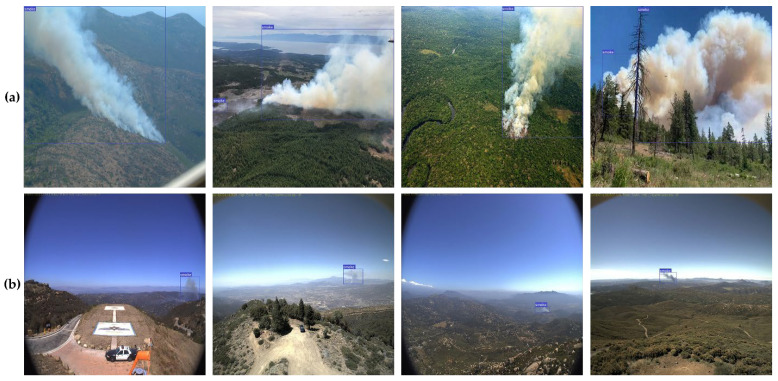
Results of the proposed wildfire smoke detection system for different forest environments: (**a**) large-sized smoke images, (**b**) small-sized smoke images.

First, we conducted a qualitative analysis of the proposed method for detecting smoke from wildfires. In the test set of our wildfire smoke dataset, we randomly selected four images for large-sized smoke and another four images for small-sized smoke. The improved YOLOv5m model yielded qualitatively similar results for images of both large—(a) and small—(b) sized smoke, as shown in Figure 10. These eight pictures show different scenes and situations as well as smoke blowing in various directions.

As shown in Figure 10, the proposed wildfire smoke detection method uses the improved YOLOv5m model, which can detect smoke in a wide range of forest scenes. We also experimented with both large- and small-sized smoke images to ensure the stability of the proposed method. Early detection of smoke is essential for wildfire prevention and suppression. If not controlled in time, even a small amount of smoke can lead to a devastating wildfire that poses a threat to human life, forest resources, and the environment. The proposed method for detecting wildfire smoke can also accurately detect relatively small regions of smoke in images.

According to experiments, the suggested method has been shown to be effective in decreasing false detections and allowing for early suppression and rapid response times, regardless of the size, direction, or shape of wildfire smoke. In most cases, when a small amount of smoke has the same color and pixel intensity values as the background, traditional visual fire detectors falsely detect it.

### 4.2. Quantitative Evaluation

As a second step, we conducted quantitative experiments using Microsoft COCO metrics, such as precision, recall, and AP (as determined by Equations (5)–(7)).

In our dataset, images have various smoke sizes, such as large and small, as well as close and long distances. To determine the most effective model, we conducted experiments with several different members of the YOLOv5 network family. YOLOv5n is a cutting-edge platform that is a reliable solution for embedded systems, the Internet of Things (IoT), and edge devices. The YOLOv5s can be used in both desktop and mobile software. YOLOv5m is the best model for a wide range of datasets and training scenarios because it achieves a good balance between speed and accuracy. For datasets where it is necessary to discover smaller objects, the YOLOv5l large model is the best option. The largest of the five models, YOLOv5x, is the most accurate. As can be seen in Table 5, larger models, such as YOLOv5l and YOLOv5x, take longer to run and have more parameters, but they also yield superior results in practice. Owing to the scope of our dataset and the nature of our research, we selected YOLOv5m. The ability to quickly detect and inform about wildfire smoke is crucial for limiting the destruction of forest ecosystems and saving lives. YOLOv5m can use deep learning to assess smoke from wildfires of varying sizes and directions. Table 5 presents a more detailed comparison of all the models, including the inference speed on the CPU and GPU and the number of parameters for a 640 × 640 image size [43].

Subsequently, we tested the enhanced YOLOv5m model for its speed on the original dataset of 6000 images and the full augmented dataset of 30,000 images to see how well it performed. As shown in Table 6, the improved YOLOv5m model performed better when using the complete augmented dataset than the original dataset, with 75.6% and 82.7%, respectively. The variation in model weight size in Table 6 does not depend on data augmentation. As far as we know, the main reason is that the training ended before the final epoch. In addition to the FP16 model, training checkpoints include an FP16 EMA and an FP32 optimizer (each model parameter has its FP32 gradient saved within the optimizer). After the last training epoch, the EMA and optimizer are removed from the final checkpoint, leaving only the FP16 model. Initially, the number of epochs was set to 600, but the training process was manually ended at 300 epochs since a predefined learning rate and accuracy were achieved. We found that if the training process ended before the final epoch, the weight size could be up to four times larger than the original model’s weight size.

We evaluated the precision of the proposed method by deploying various YOLO variants on the original wildfire smoke fire dataset (6000 images) and distinguishing the resultant precisions (Table 7). As can be seen in Table 7, the improved YOLOv5m has an average precision of 75.6% with the training set, whereas the average precisions of YOLOv5m, YOLOv4, and YOLOv3 were 73.5%, 71.3%, and 65.6%, respectively.

We also compared the average precision results of various YOLO implementations on the augmented fire dataset (30,000 images) to measure the performance of the proposed approach. The enhanced YOLOv5m model performed at the top (82.7% and 79.3% accuracy, respectively) in both the training and testing phases (Table 8). Compared with the enhanced YOLOv5m model, which achieved an average precision of 79.1% in the test set, YOLOv5m’s result was 75.4% (a difference of 3.9%). YOLOv4 and YOLOv3 were trained to average precisions of 73.5% and 78.1%, respectively.

These models were trained longer than those in the earlier experiments because of the larger number of images in the augmented dataset. With the help of data augmentation techniques, we were able to boost the overall precision of our training dataset from 75.6% to 82.7% (7.1%), and the precision of our test dataset from 72.4% to 79.3% (6.9%).

**Figure 11:**
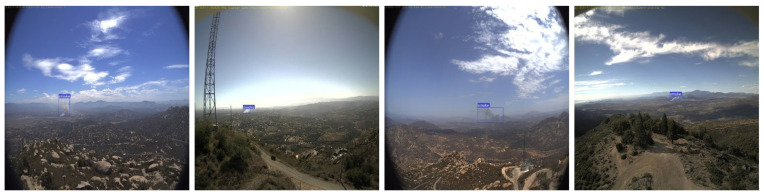
Examples of images of small-sized smoke for wildfire smoke detection dataset.

Although the average precision of the test set was 79.3%, we have researched and evaluated several recently presented methods to improve this result. Most methods proposed for detecting smoke from small wildfires in images have failed [50]. Therefore, to broaden our dataset and improve the precision of wildfire smoke detection, we collected images of small-sized smoke from wildfires. Images of smoke of relatively small sizes are shown in Figure 11. Based on [8], we combined a large-scale feature map with a feature map from a previous layer to detect small moving objects while preserving fine-grained features. Smoke pixels of varying sizes can be detected using this comprehensive feature map by combining location data from lower layers with more complex characteristics from higher ones.

To comprehensively explore the performance of the proposed method, we compared it with two-stage methods such as Fast R-CNN, Faster R-CNN+++, Mask R-CNN, Cascade R-CNN, CoupleNet, and DeNet, and one-stage methods such as RFBNet, SSD, RefineDet, DeepSmoke, EfficientDet, YOLO, YOLOv2, YOLOv3, YOLOv4, and YOLOv5 object detectors. Table 9 shows a performance comparison between the improved YOLOv5m model and the other six two-stage object detectors using the wildfire smoke dataset. To compare and assess the performance of the object detector models, we utilized the same training and testing images of smoke from the custom wildfire smoke dataset. Table 10 shows a performance comparison between the improved YOLOv5m model and the other six one-stage object detectors using the wildfire smoke dataset.

As we can observe, the improved YOLOv5m model achieved the best smoke detection performance on our wildfire smoke dataset in terms of the AP, AP50, AP75, APS, APM, and APL evaluation metrics.

### 4.3. Ablation Study

Ablation experiments are developed to confirm whether or not the SPPF+ and BiFPN described in this paper enhance the accuracy. The total number of ablation experiments is four, as follows: YOLOv5m, YOLOv5m + (SPPF+), YOLOv5m + BiFPN, and YOLOv5m + (SPPF+) + BiFPN. Experiments 2–4 were performed in the following sequence. The original YOLOv5m model was trained in the first experiment by adding only SPPF+. In the second experiment, the model was trained by adding only BiFPN to the neck part of the original YOLOv5m. In the last experiment, SPPF+ and BiFPN were added to the original YOLOv5m and trained together. Table 11 shows the comparison of the ablation experiments. Even though YOLOv5m is one of the most well-known object detection models, its results are relatively low, as demonstrated by the ablation studies (Table 11). These verify that replacing the SPPF module and modifying the PANet design in YOLOv5m to a BiFPN design can enhance the model’s performance.

## 5. Analysis of Wildfire Smoke Detection Based on Various Systems

*Thermal sensors.* As a form of thermal energy, heat is transferred from warmer to colder regions. Sensing the thermal energy transferred via convection requires either a heating element or an infrared camera. Whether this is due to a shift in the refractive index, a change in displacement, or a shift in resistance, temperature shifts can be detected by the heating element. Amplification circuits, signal conditioning, and heating elements are the three main components of thermal sensors. A thermal sensor is used to assess the level of fire-related heat within a building. There are the following three distinct types of thermal sensors: fixed temperature, increasing rate, and compensating rate sensors. The thermal detector has a minimum operating temperature or a predetermined temperature threshold. The rate of the thermal compensation sensor is triggered when the ambient air temperature rises above the set point.

*Smoke sensors.* Currently, the most prevalent and widely used fire alarm system is based on smoke sensors. Smoke can be detected in the following two different ways: photoelectric (light distribution) and ionization. To detect smoke, ionization smoke detectors use a radioactive source, whereas photoelectric detectors use a photodetector and light source. When smoke is present in the air, the particles spread light. Occlusion or light distribution can be measured using a detector. Regardless of the detection method, an alarm is produced when the signals reach a certain threshold. Its sensing principle makes fire alarms effective, responsive, and reliable.

In actual flames, ionization alarms typically react more quickly than photoelectric alarms. Compared with ionization detectors, photoelectric alarms are more reliable and sensitive in the presence of flaming fires. In summary, smoke detectors are particle detectors that are sensitive to a narrow range of particle sizes. When the signal from the smoke detector rises above a specific limit, an alarm is activated. These systems cannot always distinguish between fire-related and non-fire-related particles when they are of the same size or have the same refractive index. For instance, fire alarms are easily damaged by dust and humidity. Both photoelectric and ionization fire alarm systems suffer from high false alarm rates owing to cross-sensitivity. Additional sensors can be added to the smoke detectors to improve the reliability of fire detection.

*Vision-based fire detection.* Traditional heat, smoke, flame, and gas sensors are problematic because they take too long to reach predefined values. This is the time it takes for the particles to travel to the point sensors and trigger them. The limited coverage area is another issue. For this reason, a large number of point sensors are required to monitor large areas. When describing a fire, it is important to consider its origin, location, intensity, shape, size, growth, and dynamic texture. Traditional sensors cannot identify all these nuances. Most currently available sensors generate unnecessary alarm signals and incur additional financial expenses. The use of cameras to capture and analyze images of smoke or fire is an effective way to reduce these problems. UAV and surveillance cameras can be used instead of expensive smoke- and fire-detection sensors to further reduce costs.

*Sun-synchronous satellites.* Recently, numerous studies have attempted to detect forest wildfires using satellite imagery. This is primarily attributable to the high volume of satellite launches and declining costs. The data from three different types of multispectral imaging sensors onboard sun-synchronous satellites—the advanced very-high-resolution radiometer (AVHRR), the moderate resolution imaging spectroradiometer (MODIS), and the visible and infrared imaging radiometer suite (VIIRS)—have all been used to detect wildfires. Given its substantial similarity to clouds, haze, and other similar phenomena, smoke detection using MODIS data is a complex problem that has been addressed in multiple studies. Shukla et al. [87] proposed a multiband thresholding technique as a basis for automatic smoke detection using MODIS data. The algorithm appears to be able to distinguish smoke pixels from backgrounds with other elements, such as clouds, although it is better when the smoke is fresh and dense than when it is more dispersed. Priya et al. [88] also utilized a dataset of 534 RGB satellite images gathered from various sources, such as MODIS images available on the NASA Worldview platform and Google. A robust method for distinguishing between fire and non-fire images was developed using a CNN based on Inception v3 and transfer learning. Thresholding and nearby binary patterns were then used to isolate the areas where fires existed.

*Geostationary satellites.* The Advanced Himawari Imager (AHI) sensor of the Himawari 8 weather satellite has already been used to conduct crucial work on fire and smoke detection using satellite imagery from geostationary satellites. The Himawari 8 satellite is part of the Japan Meteorological Agency’s new-generation geostationary weather satellites. Compared to its predecessor, AHI 8 claims significantly improved radiometric, spectral, and spatial resolutions. Its primary mechanism is called the advanced baseline imager (ABI), which captures images of Earth in 16 different visible and infrared spectral bands at extremely high spatial and temporal resolutions. Recently, using information from the Himawari 8/AHI sensor, Larsen et al. [89] introduced a deep FCN for near-real-time prediction of fire smoke in satellite imagery.

*CubeSats*. Miniaturized satellites known as “CubeSats” are becoming increasingly popular in remote sensing. These satellites, which typically weigh between 1 and 10 kg and connect to the famous “CubeSat” standard, define the outer dimensions of the satellite within multiple cubic units of 10 cm × 10 cm × 10 cm. It can accommodate small technology payloads for various scientific research or commercial functions, as well as for exploring new space technologies. Owing to their specific design, CubeSats have an easier operating time in the LEO zone from a technical perspective. As of January 2020, over 1100 CubeSats had been successfully launched by various academic institutions and commercial enterprises worldwide. Shah et al. [90] introduced a system comprising a constellation of nanosatellites equipped with multispectral visible-to-infrared cameras and a ground station. This would allow all surface points on the planet to be revisited at least once per hour. To accurately estimate the thermal result of the surface, it must be captured with high resolution in both the mid- and long-wave infrared. Based on computer simulations, a multispectral infrared camera measuring incident power in two thermal infrared bands can detect a fire that spans approximately 400 square meters (mid-wave and long-wave). Because of the system’s built-in data-processing capabilities, we can issue a warning about a wildfire within 30 min and use very little network bandwidth.

## 6. Limitations and Future Research

**Figure 12:**
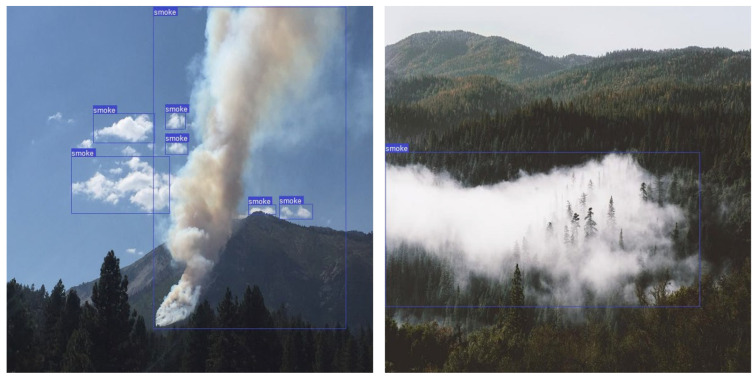
Limitations of the proposed wildfire smoke detection system.

Despite these successes, the proposed wildfire smoke detection and notification system has certain limitations. One of these is the ability to differentiate between real smoke and phenomena such as fog, haze, and clouds, which can make it appear as though smoke is present. These drawbacks are illustrated in Figure 12. These restrictions are most noticeable in scenes with dense clouds or haze, whose pixel values are close to those of a smoke plume. Our next step is to update the wildfire smoke detection system to determine the difference between different-sized clouds and different-shaped smoke so that we can better detect the source of a fire. These methods improve the ability of the model to predict the presence of smoke by effectively expanding the size of our training data and extracting better representations from the data. One technique that could broaden the scope of this field is to determine the size and shape of the smoke. The lack of consideration of the model’s potential for nighttime detection of wildfires is another potential issue. As daytime smoke detection was the focus of this investigation, this temporal variable was omitted. Our research suggests that smoke detectors are less reliable in the dark than fire detectors. Agirman et al. [91] proposed a method that incorporates both the spatial and temporal behavior of a nighttime wildfire by using a CNN+RNN-based network and a bidirectional long short-term memory network to detect fire. Note that this study covered only the AI server part of the wildfire-smoke detection and notification system.

Future work will focus on addressing the model’s limitation of having a high number of false positives in challenging scenarios, such as low-altitude cloud cover and haze. Incorporating fire location, date, and weather data from historical fire records can improve predictions because fires tend to occur in similar locations and conditions during specific months. Another drawback of the proposed approach is its incompatibility with edge devices. However, we hope to address this in future work by reducing the model size without compromising prediction accuracy. It is feasible to create a model that is better suited for edge computing by using distillation to train a smaller deep network, such as YOLOv5n, to achieve the same level of performance as our current model.

## 7. Conclusions

Several researchers have attempted to employ a CNN-based deep learning model to enhance wildfire smoke detection systems as remote camera sensing technology has advanced. Collecting sufficient image data for training models in wildfire detection is challenging, leading to data imbalance or overfitting issues that decrease the model’s performance. This study developed an early wildfire smoke detection and notification system using an improved YOLOv5m model and a wildfire image dataset.

Initially, the classification error was decreased using the K-mean++ method to enhance the anchor box clustering of the backbone part. Second, the SPPF layer of the backbone part was upgraded to SPPF+ to better concentrate the smoke from small wildfires. A Bi-FPN module was implemented as a third step to fine-tune the neck and achieve a more precise fusion of features across multiple scales. Finally, during training, network pruning and transfer learning techniques were used to enhance the network architecture, detection accuracy, and speed. The proposed wildfire smoke detection method was trained using smoke images, including various wildfire smoke scenes, and conducted on an AI server. We collected a wildfire smoke dataset that included 6000 smoke and non-smoke images for model training and testing. We compared the proposed system to other popular two-stage object detectors in an experiment to evaluate the qualitative and quantitative performance. Based on the experimental results and evaluation, it was concluded that the enhanced YOLOv5m model is robust and outperforms other methods in the training and testing steps, with 82.7% and 81.5% AP_50_, respectively, on the custom smoke image dataset.

## Figures and Tables

**Table 1:** Wildfire smoke dataset and its sources.

Name of Dataset	Number of Smoke Images (3285)				Number of Non-Smoke Images (2715)				Total
	Kaggle	Bing	Google	Flickr	Kaggle	Bing	Google	Flickr	
Wildfire Smoke	2580	250	300	155	2460	100	105	50	6000

**Table 2:** Number of training and testing images in wildfire smoke dataset.

Wildfire Smoke Detection Dataset	Number of Training Images			Number of Testing Images	Total
	Original Images	Image Rotation	Image Flipping	Original Images	
Wildfire smoke images	2628	5256	7884	657	16,425
Non-smoke images	2172	4344	6516	543	13,575
Total	4800	9600	14,400	1200	30,000

**Table 3:** Microsoft’s COCO benchmarks—widely used in object detection tasks to evaluate precision and recall at various levels.

AP	AP_50_	AP at IoU = 0.5
AP	AP_75_	AP at IoU = 0.75
AP at various levels	AP_S_	AP_0.5_ for small regions: area < 32^2^
	AP_M_	AP_0.5_ for medium regions: 32^2^ < area < 96^2^
	AP_L_	AP_0.5_ for large regions: area > 96^2^

**Table 4:** The specifications of the AI server.

Hardware Parts	Detailed Specifications
Storage	SSD: 512 GB HDD: 2 TB (2 are installed)
Motherboard	ASUS PRIME Z390-A
Operating System	Ubuntu Desktop
Graphic Processing Unit	GeForce RTX 2080 Ti 11 GB (2 are installed)
Central Processing Unit	Intel Core 9 Gen i7-9700k (4.90 GHz)
Random Access Memory	DDR4 16 GB (4 are installed)
Local Area Network	Internal port—10/100 Mbps External port—10/100 Mbps
Power	1000 W (+12 V Single Rail)

**Table 5:** Description of YOLOv5 models.

Models	AP 0.5:0.95	AP 0.5	Speed CPU (ms)	Speed GPU (ms)	Parameters (million)	FLOPS (b)	Iteration number
YOLOv5n	28.0	45.7	45	6.3	1.9	4.5	300
YOLOv5s	37.4	56.8	98	6.4	7.2	16.5	
YOLOv5m	45.4	64.1	224	8.2	21.2	49.0	
YOLOv5l	49.0	67.3	430	10.1	46.5	109.1	
YOLOv5x	50.7	68.9	766	12.1	86.7	205.7	

**Table 6:** Precision of improved YOLOv5m during training process.

Model	Input Size	Training (AP_50_)		Training Time		Weight Size	
		Before DA	After DA	Before DA	After DA	Before DA	After DA
Improved YOLOv5m	640 × 640	75.6	82.7	46 h	85 h	68 MB	93 MB

**Table 7:** Average precision of YOLO models with original wildfire smoke images.

Models	Training Input Size	Training (AP_50_)	Testing Input Size	Testing (AP_50_)	Iteration Number
YOLOv3 [26]	416 × 416	65.6	640 × 640	63.5	300
YOLOv4 [27]	608 × 608	71.3		68.6	
YOLOv5m [42]	640 × 640	73.5		70.8	
Improved YOLOv5m	640 × 640	75.6		72.4	

**Table 8:** Average precision of YOLO models with augmented wildfire smoke images.

Models	Training Input Size	Training (AP_50_)	Testing Input Size	Testing (AP_50_)	Iteration Number
YOLOv3 [26]	416 × 416	73.5	640 × 640	69.8	300
YOLOv4 [27]	608 × 608	78.1		73.9	
YOLOv5m [42]	640 × 640	79.6		75.4	
Improved YOLOv5m	640 × 640	82.7		79.3	

**Table 9:** The proposed method versus other two-stage object detectors on the custom wildfire smoke dataset test-set. Improved YOLOv5m achieves results that are competitive with two-stage object detectors.

Model	Backbone	AP	AP_50_	AP_75_	AP_S_	AP_M_	AP_L_
DeNet [75]	ResNet-101	55.4	63.7	58.2	46.3	55.8	61.5
CoupleNet [76]	ResNet-101	58.6	65.2	60.7	48.6	58.4	63.7
Fast R-CNN [77]	ResNet-101	61.5	68.3	62.4	51.8	60.4	66.1
Faster R-CNN [78]	ResNet-101	63.7	70.6	65.7	54.3	62.6	68.2
Mask R-CNN [79]	ResNet-101	67.5	75.8	70.9	59.4	66.3	73.1
Cascade R-CNN [80]	ResNet-101	70.2	78.4	74.3	62.8	69.1	75.6
Improved YOLOv5m	CSPDarknet-53	**73.6**	**81.5**	**76.3**	**65.7**	**72.4**	**78.6**

**Table 10:** The proposed method versus other one-stage object detectors on the custom wildfire smoke dataset test-set. Improved YOLOv5m outperforms all one-stage object detectors.

Model	Backbone	AP	AP_50_	AP_75_	AP_S_	AP_M_	AP_L_
RFBNet [81]	VGG-16	62.4	68.5	63.7	51.2	59.6	72.8
SSD [82]	VGG-16	63.7	71.3	65.8	54.7	63.1	76.4
RefineDet [83]	VGG-16	68.3	75.8	70.6	59.8	66.3	81.7
EfficientDet [63]	EfficientNet	70.6	77.4	73.1	62.5	69.0	82.9
DeepSmoke [84]	EfficientNet	71.4	78.6	74.5	63.4	70.5	85.3
YOLO [85]	GoogleNet	56.3	62.6	54.8	46.2	55.7	68.1
YOLOv2 [86]	Darknet-19	64.8	71.7	65.2	55.6	64.3	75.4
YOLOv3 [26]	Darknet-53	67.2	75.4	68.5	59.1	66.7	78.6
YOLOv4 [27]	CSPDarknet-53	69.7	77.5	71.6	60.4	68.2	81.8
YOLOv5m [42]	CSPDarknet-53	70.9	78.2	72.4	62.8	69.5	83.6
Improved YOLOv5m	CSPDarknet-53	**73.6**	**81.5**	**76.3**	**65.7**	**72.4**	**87.2**

**Table 11:** Comparison results of the ablation experiments.

Model	AP	AP_50_	AP_75_	AP_S_	AP_M_	AP_L_
YOLOv5m	70.9	78.2	72.4	62.8	69.5	83.6
YOLOv5m+SPPF+	71.6	78.5	73.2	63.7	70.4	84.7
YOLOv5m+BiFPN	72.4	79.2	74.5	64.6	71.3	86.1
YOLOv5m+(SPPF+)+BiFPN	**73.6**	**81.5**	**76.3**	**65.7**	**72.4**	**87.2**

## Data Availability

Not applicable.
